# Delayed presentation of congenital diaphragmatic hernia with bowel obstruction in an 8-year-old, diagnostic value of chest X-ray as the sole imaging modality: a case report and literature review

**DOI:** 10.1097/RC9.0000000000000263

**Published:** 2026-02-12

**Authors:** Nelton Rodrick Thobias

**Affiliations:** Faculty of Medicine, Kilimanjaro Christian Medical University College, Moshi, Kilimanjaro region, Tanzania; Department of Emergency Medicine, Mawenzi Regional Referral Hospital, Moshi, Kilimanjaro region, Tanzania

**Keywords:** chest-abdominal X-ray, congenital diaphragmatic hernia, intestinal obstruction, laparotomy

## Abstract

**Introduction:**

Congenital diaphragmatic hernia (CDH) manifests as a result of incomplete closure of the diaphragm muscles during embryological development. Most cases are diagnosed during the antenatal period or may manifest during the first week of life. In this case, we have reported a child patient with a late-presenting left congenital diaphragmatic hernia with bowel obstruction diagnosed by an X-ray alone.

**Presentation of case:**

An 8-year-old boy presented with severe generalized abdominal pain, associated with vomiting, constipation, and difficulty in breathing. On examination, the child had a scaphoid abdomen with no audible bowel sounds. His chest had no air entry on the left side, audible bowel sounds on the left, mediastinal shift to the right side of the chest, and muffled heart sounds on the left side of the chest. Chest-abdominal X-ray, erect view, revealed multiple bowel loops in the left hemithorax with loss of the left hemithorax dome, cardiac silhouette shifted more to the right side of the chest. Emergency laparotomy was done with good post-operative recovery.

**Discussion:**

Delayed (late-presenting) congenital diaphragmatic hernia (CDH) is an uncommon clinical entity that poses diagnostic and therapeutic challenges because it often presents outside the neonatal period with heterogeneous respiratory or gastrointestinal symptoms and is frequently misdiagnosed.

**Conclusion:**

This report reinforces the need for clinicians – particularly in resource-limited settings – to consider CDH in older children with compatible clinical and radiographic features and demonstrates that timely surgery based on careful chest radiograph interpretation can lead to good outcomes.

## Introduction

Congenital diaphragmatic hernia (CDH) manifests as a result of incomplete closure of the diaphragm muscles during embryological development, and most cases are diagnosed during the antenatal period or may manifest during the first week of life^[^1,2^]^. CDH is a rare condition occurring in < 1–5:10 000 births^[^3^]^. Delayed presentation is infrequent, and clinical presentations differ from person to person; some may present with mild respiratory symptoms, while others may present with gastrointestinal symptoms like those of intestinal obstruction^[^2^]^.

**HIGHLIGHTS**

- Congenital diaphragmatic hernia (CDH) manifests as a result of incomplete closure of the diaphragm muscles during embryological development.
- An 8-year-old boy presented with severe generalized abdominal pain, associated with vomiting, constipation, and difficulty in breathing. On examination, the child had a scaphoid abdomen with no audible bowel sounds. His chest had no air entry on the left side, audible bowel sounds on the left, mediastinal shift to the right side of the chest, with muffled heart sounds on the left side of the chest.
- Chest-abdominal X-ray erect view revealed loops of small bowel in the left hemithorax with loss of left hemithorax dome, cardiac silhouette shifted more to the right side of the chest.
- Emergency laparotomy was done with good post op recovery.

CDH can be misdiagnosed and mistreated as a case of tension pneumothorax for patients with acute presentations first contact at the emergency department^[^4^]^. Both late-presenting CDH and tension pneumothorax can produce acute respiratory distress and a mediastinal shift; however, CDH is usually accompanied by gastrointestinal symptoms such as vomiting, constipation, and abdominal pain, which are not typical clinical manifestations in tension pneumothorax^[^4^]^.

In this case, we have reported a child patient with a late-presenting left congenital diaphragmatic hernia with bowel obstruction diagnosed by an X-ray alone. This case report has been reported in line with the SCARE checklist^[^5^]^.

## Presentation of case

**Figure 1.**
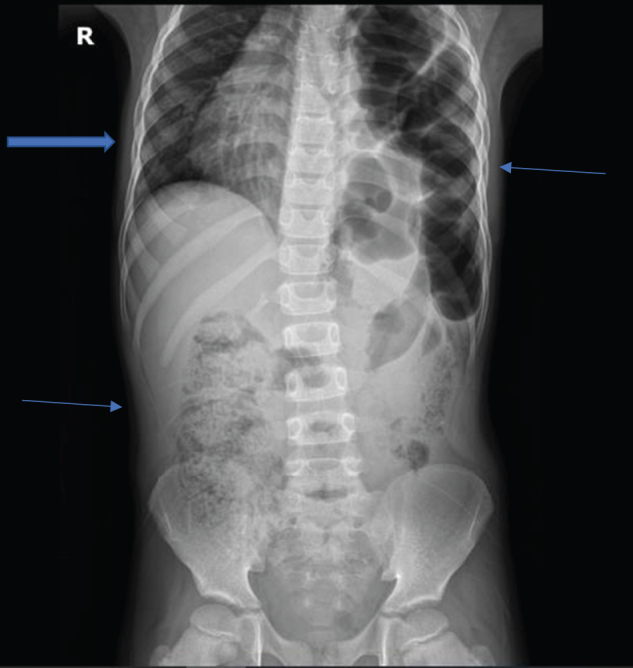
Chest-abdominal X-ray erect view showing loops of large bowel in the left hemithorax with loss of left hemithorax dome (thin blue arrow on the upper left side), fecal material in the ascending colon (thin blue arrow on the lower right side), and mediastinal shift to the right with pseudodextrocardia (thick blue block arrow on the upper right side).

**Figure 2.**
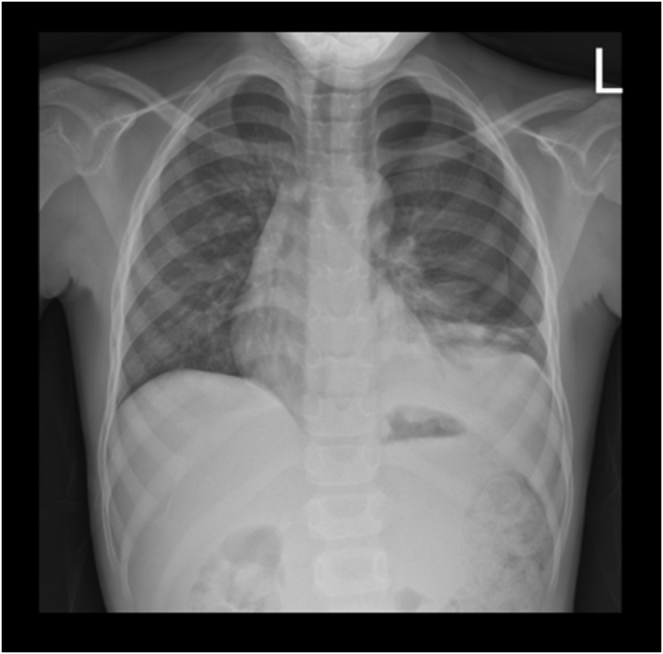
A control chest X-ray done after surgical repair of the diaphragmatic defect, showing no bowel loops in the left hemithorax.

An 8-year-old boy patient who was a referral case from HAI district hospital, the reason for referral was an abdominal CT-scan. The informant was his father. He reported that the child had a complaint of severe generalized abdominal pain, cramping in nature, for 5 days, which was associated with non-projectile vomiting of everything, not passing stool for 4 days, and difficulty in breathing, exacerbated when lying down leaning on the right side, while improving when lying down leaning on the left side. However, there was no history of abdominal distension, passing loose stool, or cough. On physical examination, the child appeared well-built and comfortable; he was afebrile, with a normal blood pressure. He had a scaphoid abdomen with no audible bowel sounds. His chest had no air entry on the left side, audible bowel sounds on the left, mediastinal shift to the right side of the chest, apex beat was on the 5th intercostal space right midclavicular line, with muffled heart sounds on the left side of the chest. Imaging investigation; Chest-abdominal X-ray erect view revealed multiple bowel loops in the left hemithorax with loss of left hemithorax dome, cardiac silhouette shifted more to the right side of the chest, fecal material in the ascending colon, with the impression of left diaphragmatic hernia with loops of large bowel in the left hemithorax (Fig. 1). The surgical department was consulted, and the patient underwent emergency laparotomy. Intra-operative findings: Inspection of the bowels was done, starting from the small bowel at the ligament of Treitz to the terminal ileum. The ascending, transverse colon, together with the spleen, was all found herniated into the left thoracic cavity through a paraesophageal diaphragmatic defect of approximately 6 cm × 4 cm. Reduction of the abdominal viscera was done by gentle traction. Inspection of the bowels and spleen was done; the bowels and spleen were viable and intact. The diaphragmatic defect was repaired, then realignment of the spleen and bowels was done in anatomical position, and then the abdomen was closed in layers, with the patient free from sedation, was transferred to the ward for recovery. The patient was put on intravenous antibiotics (ceftriaxone and metronidazole), 3 days later control chest X-ray was done, which showed no bowel loops in the left hemithorax (Fig. 2). The patient was discharged.

## Discussion

Delayed (late-presenting) congenital diaphragmatic hernia (CDH) is an uncommon clinical entity that poses diagnostic and therapeutic challenges because it often presents outside the neonatal period with heterogeneous respiratory or gastrointestinal symptoms and is frequently misdiagnosed^[^6,7^]^. Most large series and reviews emphasize that the majority of CDH cases are detected prenatally or in the neonatal period; however, a minority present later in childhood or even adulthood, often after an intervening period of normal health^[^6,8^]^. Our patient – a previously well 8-year-old who presented with acute bowel obstruction secondary to a left posterolateral (Bochdalek) defect – is a representative example of this late-presenting spectrum and illustrates several clinically important points.

Late-presenting CDH comprises a small fraction of all CDH cases but shows a wide clinical spectrum ranging from incidental radiographic findings to life-threatening intestinal strangulation or respiratory compromise^[^6,9^]^. Bagłaj’s multi-institutional review and other series estimate that significant numbers of children with late presentation have been reported in case series and literature reviews, emphasizing both the rarity and the variable presentation^[^6^]^. The literature documents presentations with chronic cough, recurrent chest infections, failure to thrive, acute abdominal symptoms due to bowel obstruction or volvulus, and even isolated gastrointestinal complaints that led to delayed diagnosis^[^7,10,11^]^. Our patient’s presentation with frank bowel obstruction and the need for urgent surgery places this case among the subset of late CDH presentations that are associated with higher acute morbidity if diagnosis is delayed^[^6,10^]^.

Chest radiography remains the frontline investigation in many settings and can show pathognomonic features of CDH (air-filled bowel loops in the hemithorax, mediastinal shift, or absence of a diaphragmatic contour), but findings may be subtle or masquerade as common conditions (pneumonia, pleural effusion, pneumothorax) leading to misinterpretation and potentially harmful interventions (e.g., chest tube placement)^[^12,13^]^. Several case series and radiologic reviews underline that plain chest X-ray will correctly suggest the diagnosis in many – but not all – cases, and further imaging (contrast study, ultrasound, or CT) is often recommended to confirm anatomy and plan operative approach when available^[^9,11,14^]^. In low-resource environments or when families cannot afford CT, however, a high index of suspicion combined with careful radiographic interpretation may allow a safe and accurate diagnosis; there are multiple reports documenting successful operative management after diagnosis based on chest radiography alone^[^15–17^]^. Our case is important in this regard: the diagnosis was made on a single chest X-ray because CT was unobtainable for financial reasons, and subsequent intraoperative findings confirmed the radiographic interpretation. This emphasizes that, while cross-sectional imaging is desirable, chest X-ray can be diagnostic and be used to guide timely surgery when other modalities are unavailable.

Multiple recent case reports and reviews describe children and adults diagnosed with late CDH, with several emphasizing diagnostic delays and pitfalls^[^7,9,10,15,18^]^. Kim *et al* (2013) and later case reports highlight the variable imaging appearances and the danger of misdiagnosing CDH as pulmonary pathology^[^9,11^]^. More recent reports continue to document presentations similar to ours – older children presenting with gastrointestinal obstruction and successful repair after diagnosis from radiographs – reinforcing that late presentation with acute abdomen remains an important, if uncommon, clinical scenario^[^8,16,19^]^. What distinguishes our case from many published reports is the combination of (1) relatively advanced pediatric age (8 years), (2) acute mechanical bowel obstruction as the presenting problem, and (3) reliance on a single chest radiograph as the sole imaging modality due to financial constraints, with a favorable surgical outcome. Although similar elements are scattered through the literature, the combination is less commonly emphasized in large reviews and provides a concrete message for clinicians working in resource-limited settings.

Surgical repair remains the definitive therapy for symptomatic late CDH; the approach (open vs thoracoscopic/laparoscopic) and need for prosthetic patch depend on size and chronicity of the defect and intraoperative findings^[^7,20^]^. Outcomes for late presenters are generally favorable if diagnosis and repair are timely; the major risks relate to bowel ischemia/strangulation when hollow viscera herniate, and to pulmonary compromise when the thoracic cavity is occupied by abdominal contents^[^6,7^]^. Our patient’s uncomplicated postoperative course aligns with series that report good outcomes after prompt operative management^[^6,21^]^. The literature also supports that delayed diagnosis can lead to higher rates of preoperative complications and, in some cases, iatrogenic harm from incorrect interventions (e.g., chest tube for presumed pneumothorax)^[^7,11,13^]^.

This case has three practical lessons:

Maintain diagnostic suspicion for CDH outside the neonatal period. Recurrent or atypical respiratory symptoms, unexplained gastrointestinal complaints, or acute abdominal signs – especially when chest X-ray shows an abnormal hemithorax – should prompt consideration of late CDH in the differential diagnosis. Several authoritative reviews and series sound this same warning^[^6,7,9^]^.

Chest radiography can be diagnostic and sufficient to proceed to surgery when further imaging is not feasible. While CT or contrast studies provide useful anatomic detail, chest X-ray may be the only available study in resource-limited settings; when interpreted carefully (air-filled bowel in thorax, nasogastric tube curving into hemithorax, mediastinal shift), it can justify timely operative intervention and avoid potentially catastrophic delay^[^12,14,16,17^]^. Our experience corroborates similar case reports from limited-resource environments^[^15,17^]^.

Avoid premature invasive thoracic procedures when radiographic features suggest intrathoracic bowel. Awareness of the radiographic mimicry between CDH and other thoracic conditions must be emphasized to prevent iatrogenic injury; this precaution is strongly recommended across imaging and surgical literature^[^11,13^]^.

## Conclusion

Although late-presenting CDH has been described previously, the combination of an 8-year-old child presenting with acute bowel obstruction and diagnosis made by chest X-ray alone (due to socioeconomic constraints) has clear educational value. This report reinforces the need for clinicians – particularly in resource-limited settings – to consider CDH in older children with compatible clinical and radiographic features and demonstrates that timely surgery based on careful chest radiograph interpretation can lead to good outcomes.

## Data Availability

There were no generated data in this study.
